# Incidence of sick leave and disability pension in adults with musculoskeletal pain and co-occurring long-term conditions: data from the Norwegian HUNT study and national registries

**DOI:** 10.1186/s12891-024-07405-1

**Published:** 2024-04-08

**Authors:** Anna Marcuzzi, Paul Jarle Mork, Lene Aasdahl, Eivind Skarpsno, Karoline Moe, Tom Ivar Lund Nilsen

**Affiliations:** 1 Department of Public Health and Nursing, Norwegian University of Science and Technology (NTNU), Trondheim, Norway; 2 Department of Physical Medicine and Rehabilitation, St. Olavs Hospital, Trondheim, Norway; 3 Department of Neuromedicine and Movement Science, Norwegian University of Science and Technology (NTNU), Trondheim, Norway; 4 Unicare Helsefort Rehabilitation Center, Rissa, Norway

**Keywords:** Musculoskeletal pain, Work disability, Sick leave, Multimorbidity

## Abstract

**Background:**

Musculoskeletal pain is one of the leading causes of work productivity loss. Long-term conditions (LTCs) commonly occur alongside musculoskeletal pain. However, the incidence of sick leave and disability pension according to LTC status in people with musculoskeletal pain has not been previously described.

**Methods:**

Working-age participants (20–65 years) with persistent musculoskeletal pain who participated in the HUNT3 Study (1995–97) were included. Twenty-five LTCs were classified into 8 LTC groups according to the International Classification of Diseases version 11. Data on sickness and disability benefits were obtained from the National Insurance Database and linked to the HUNT3 data using participants’ personal identification number. Age-adjusted incidence rates (IRs) (per 10,000 person-years) and hazard ratios (HRs) of sick leave during 5-year follow-up and disability pension during ~ 25-year follow-up were estimated with 95% confidence intervals (CIs) and presented according to LTC status.

**Results:**

Overall, 11,080 participants with musculoskeletal pain were included. Of those, 32% reported one LTC and 45% reported ≥ 2 LTCs. During the follow up period, 1,312 participants (12%) received disability pension due to musculoskeletal conditions. The IR of sick leave and disability pension due to musculoskeletal conditions increased with number of LTCs. Specifically, the IR of sick leave was 720 (95% CI 672 to 768) in participants without any LTCs and 968 (95% CI 927 to 1,009) if they had ≥ 2 LTCs. The IRs of disability pension were 87 (95% CI 75 to 98) and 167 (95% CI 154 to 179) among those with no LTCs and ≥ 2 LTCs, respectively. The incidence of sick leave and disability pension due to musculoskeletal conditions was largely similar across LTCs, although the incidence of disability pension was somewhat higher among people with sleep disorders (IR: 223, 95% CI 194 to 252).

**Conclusions:**

Among people with persistent musculoskeletal pain, the incidence of prematurely leaving the work force due to musculoskeletal conditions was twice as high for those with multiple LTCs compared to those without any LTCs. This was largely irrespective of the type of LTC, indicating that the number of LTCs are an important feature when evaluating work participation among people with musculoskeletal pain.

**Supplementary Information:**

The online version contains supplementary material available at 10.1186/s12891-024-07405-1.

## Introduction

Musculoskeletal pain is highly prevalent and among the leading causes of years lived with disability globally [1]. Besides the suffering of affected individuals, musculoskeletal pain is also one of the main reasons for reduced work ability and productivity loss [2]. In Norway, musculoskeletal conditions account for almost half of all long-term sick leaves and around one-third of all disability pensions [3], with an estimated annual cost of 1.2% of the gross domestic product [4], thus representing a major societal and economic challenge.

Previous research on prognostic factors for work ability have primarily focused on specific musculoskeletal pain conditions (e.g., low back pain) and mostly considered musculoskeletal pain as a single disease entity [5]. However, other long-term conditions (LTCs) commonly occur alongside musculoskeletal pain [6]. The presence of multimorbidity (i.e., the co-occurrence of two or more LTCs) can complicate clinical management [7, 8] and has been associated with higher subjective burden and increased healthcare use in people with musculoskeletal pain [9]. Nevertheless, knowledge on work participation associated with multimorbidity in people with musculoskeletal pain is scarce and limited to the combination with psychological morbidities. For example, people with musculoskeletal pain and co-occurring mental health conditions (e.g., anxiety, depression) had greater work loss, i.e., sickness absence days, as well as more than twice the risk of disability pension compared to each condition occurring alone [10–13]. Greater knowledge on the burden of sick leave and disability pension associated with specific co-occurring LTCs among people with persistent musculoskeletal pain can help to inform more effective interventions and plan for more efficient health care services, e.g., adopting a stratified care approach. Therefore, the aim of this study was to describe the incidence of sick leave and disability pension according to the number and type of co-occurring LTCs in people with persistent musculoskeletal pain.

## Methods

### Study population

This study uses data from the third survey of the population-based HUNT Study in Norway (HUNT3, 2006–2008) along with data on sickness and disability benefits obtained from the National Insurance Database. All 93,860 inhabitants aged 20 years or more residing in the geographical region of Nord-Trøndelag were invited to the HUNT3 Study, and 54% accepted the invitation to participate. A description of the HUNT study procedures and participation rates have been published elsewhere [14], and an overview of the various questionnaires can be found at https://www.ntnu.edu/hunt/data/que. This study was registered in OSF registry [15] as part of a larger project.

**Fig. 1:**
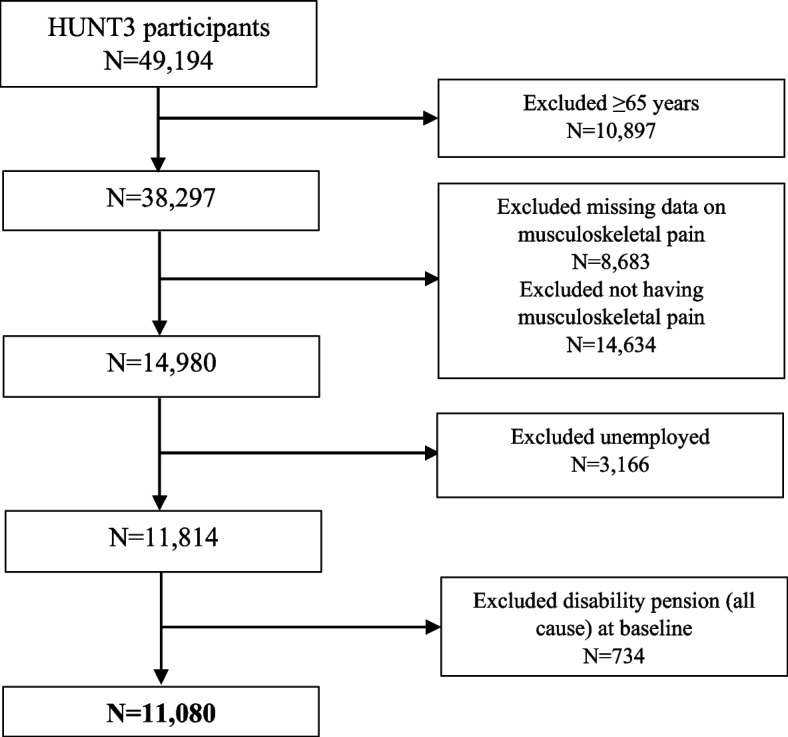
Participants’ selection into the study

The selection of participants into the current study is shown in Fig. 1. Exclusion criteria were age 65 years or older, not reporting musculoskeletal pain, and currently not working, i.e., unemployed (based on self-report from the HUNT3 survey) or having received disability benefits before or up to the date of participation in the HUNT3 survey. Participants aged 65 years or older were excluded as potentially not being able to obtain a disability pension being too close to the Norwegian statutory age of retirement, i.e., 67 years. Overall, the study included 11,080 participants reporting musculoskeletal pain lasting for at least three consecutive months during the past year.

### Sickness and disability benefits

Data on sickness and disability benefits were obtained from the National Insurance Database between 1 January 2000 and 31 December 2021 and linked to the HUNT3 data using the personal identification number held by all residents in Norway. In Norway, medically certified sick leave is compensated with 100% coverage up to 12 months, whereby the employer covers the first 16 days, and the rest is covered by the Norwegian Welfare and Labor Administration. After 12 months of sick leave, it is possible to apply for more long-term medical benefits such as work assessment allowance and disability pension. Permanent medical disability benefits can be granted to people aged between 18 and 67 years if the ability to work is permanently reduced by at least 50% due to illness or injury and after appropriate treatment and vocational rehabilitation measures have been tried. Primary diagnoses for both sickness and disability benefits were classified according to the International Classification of Diseases (ICD) versions 9 and 10 and the International Classification of Primary Care (ICPC) version 1 and 2. Sickness and disability benefits due to musculoskeletal conditions corresponded to codes M00-M99 of ICD and codes L00-L99 of ICPC. Sickness and disability benefits due to all causes corresponded to codes for all diagnoses including those due to musculoskeletal conditions.

### Persistent musculoskeletal pain

Information about musculoskeletal pain was retrieved using the Standardised Nordic Questionnaire [16] within the HUNT3 survey. Participants who reported having persistent musculoskeletal pain by ticking ‘yes’ on the following question “During the last year, have you had pain and/or stiffness in muscles or joints that lasted for at least 3 consecutive months?” were included in the study. Those with missing data on this question were excluded.

Participants who reported musculoskeletal pain were asked to indicate the affected body area(s), i.e., neck, shoulders, upper back, elbows, low back, hips, wrists/hands, knees, and ankles/feet. For descriptive purposes, participants were categorised based on the number of pain sites, regardless of the pain location as ‘1–2 pain sites’, ‘3–4 pain sites’, and ‘5 or more pain sites.’

### Long-term conditions

LTCs were identified using data from the HUNT3 survey and were categorised as reported previously [17]. An overview of the questions and measurements used to define LTCs is provided in Supplementary File 1. Overall, 25 LTCs were identified and categorised into 8 LTC groups corresponding to different body organ systems according to the 11th revision of the International Classification of Diseases (ICD-11) (Table 1). As such, each LTC group could include one or more LTCs within the same body organ system. Participants were categorised into three groups: ‘No LTC’, ‘1 LTC’, ‘ ≥ 2 LTCs’. In addition, participants were categorised as having a specific LTC co-occurring with musculoskeletal pain (e.g., metabolic conditions, mental health conditions, etc.).

**Table 1:** Grouping of long-term conditions according to the International Classification of Diseases (ICD-11)

ICD-11 Chapters	Long-term conditions included
Chapter V	
**Endocrine, nutritional, or metabolic diseases**	• Diabetes
	• Obesity
	• Hypercholesterolemia
	• Hyperthyroidism
	• Hypothyroidism
Chapter VI	
**Mental or behavioural disorders**	• Anxiety
	• Depression
	• Psychiatric problems
	• Alcohol use disorders
Chapter VII	
**Sleep–wake disorders**	• Insomnia symptoms
	• Apnoea
Chapter VIII	
** Diseases of the nervous systems**	• Chronic headache
	• Migraine
	• Epilepsy
Chapter XI	
**Diseases of the circulatory system**	• Hypertension
	• Angina pectoris
	• Myocardial infarction
	• Heart failure
	• Other heart diseases
Chapter XII	
**Diseases of the respiratory system**	• Chronic bronchitis, emphysema or chronic obstructive pulmonary diseases
	• Asthma
Chapter XIII	
**Diseases of the digestive system**	• Irritable bowel syndrome
	• Gastro-oesophageal reflux disease
Chapter XIV	
**Diseases of the skin**	• Hand eczema
	• Psoriasis

### Missing data

Participants who did not fill out a response to any questions used to categorise an LTC were classified as not having the condition. An overview of the proportion of missing data for each LTC is provided in Supplementary File 1. Missingness did not exceed 8.6% (alcohol use disorder).

### Follow up

Sick leave was registered from the date of participation in the HUNT3 survey and ended after five years. Participants contributed person-years when they were at risk for sick leave, i.e., the period when they were on sick leave due to a musculoskeletal condition or other causes was censored (discontinued recurrent events). Participants were censored at the date of death or at the date of receiving disability pension. An event was defined as sick leave due to a musculoskeletal diagnosis lasting ≥ 31 days. A duration of ≥ 31 days was chosen to reflect long-term sick leave.

For disability pension, participants were followed from their participation in the HUNT3 survey until the date they received partial or full disability pension due to a musculoskeletal condition, or else, until the date of end of follow up, 31 December 2021. In analyses of disability pension due to musculoskeletal conditions, participants were censored at the date of death or at the date of receiving disability pension due to other causes.

### Statistical analysis

We estimated incidence rates (IRs) of sick leave (including recurrent events) and disability pension per 10,000 person-years with 95% confidence intervals (CIs) in participants according to number of LTCs (i.e., no LTC, 1 LTC, and ≥ 2 LTCs) and categories of specific LTCs. Age-adjusted IRs were estimated using Poisson regression controlling for age (years) as a continuous variable (i.e., the Stata command *poisson*). Cox proportional hazard regression (i.e., *stcox*) estimated age-adjusted hazard ratios (HRs) with 95% CIs for the association of LTCs with sick leave and disability pension. The model accounted for dependency in the observations (i.e., recurrent events) using a robust variance estimator (i.e., *vce robust*). The person-year denominator for the sick leave incidence rate did not include time periods where participants had sick leave due to other causes. In sensitivity analysis we also estimated risk of a first sick leave episode, i.e., participants contributed person-years until the first sick leave event due to musculoskeletal conditions.

As the combination of LTCs as well as the incidence of sick leave and disability pension might differ across sociodemographic variables, we reported IRs and HRs for sick leave and disability pension associated with number of LTCs by age (≤ 45 years, > 45 years), sex (female, male), and educational level (≤ 12 years, > 12 years) in supplementary analyses. Finally, cumulative sick leave days due to musculoskeletal conditions and due to all causes were reported descriptively by number and type of co-occurring LTCs, i.e., median duration of sick leave episodes with interquartile range (IQR), average number of sick leave days with 95% CI.

Statistical analyses were performed using built-in commands in Stata 18.0 MP (StataCorp LLC, Stata Statistical Software: release 18. College Station, TX, USA).

## Results

Table 2 presents baseline characteristics of the study sample according to the number of LTCs. The proportion of people with musculoskeletal pain reporting no LTC was 23.4%, while 31.6% and 45.1% reported 1 LTC and 2 or more LTCs, respectively. The distribution of demographic characteristics, i.e., age, sex and education were largely similar between groups with and without LTCs, whereas the proportion reporting good/very good self-reported health was lower among those with ≥ 2 LTCs. The latter group also reported higher number of pain sites compared to those with 1 or no LTC.

**Table 2:** Baseline characteristics of participants with musculoskeletal pain stratified by number of long-term conditions

	All	No LTC	1 LTC	≥ 2 LTCs
Participants, n (%)	11,080 (100.0)	2,588 (23.4)	3,496 (31.6)	4,996 (45.1)
Age, years	47.9 (10.1)	47.8 (10.1)	47.8 (10.2)	48.0 (10.1)
Females, n (%)	6,494 (58.6)	1,478 (57.1)	1,994 (57.0)	3,022 (60.5)
Higher education^a^, n (%)	3,234 (29.2)	865 (33.4)	1,058 (30.3)	1,311 (26.2)
Self-reported good/very good health, n (%)	7,933 (71.6)	2,174 (84.0)	2,716 (77.7)	3,043 (60.9)
No. of pain sites, n (%)				
1–2	5,400 (48.7)	1,569 (60.6)	1,844 (52.8)	1,987 (39.7)
3–4	3,679 (33.2)	744 (28.8)	1,130 (32.3)	1,805 (36.1)
5-9	2,001 (18.1)	275 (10.6)	522 (14.9)	1,204 (24.1)

*LTC* Long-term condition

^a^ ≥ 12 years education

**Fig. 2:**
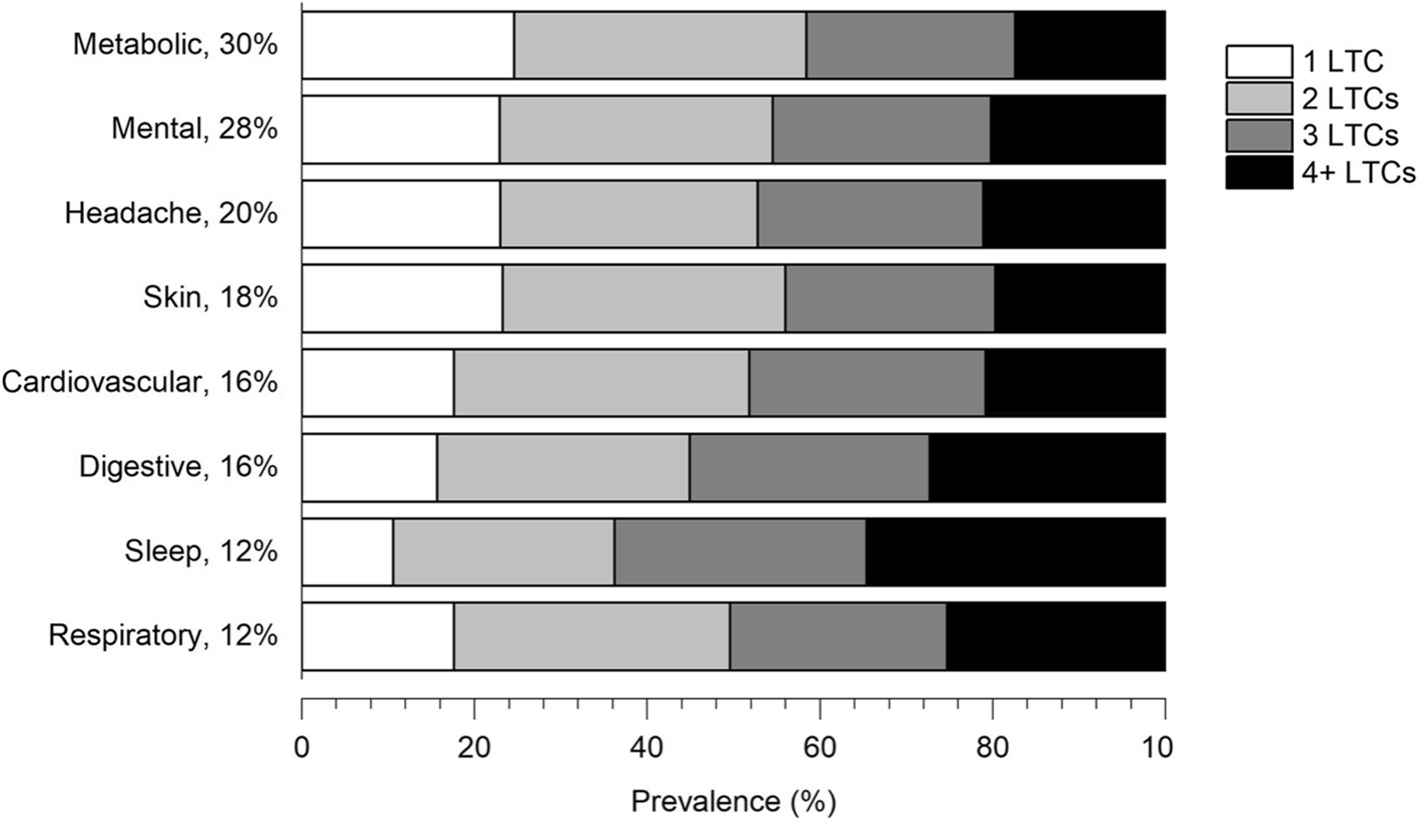
Prevalence of number of long-term conditions within each long-term condition group. Percentages specified besides each LTC represent the prevalence of the LTC in the sample

In this sample of people with musculoskeletal pain, metabolic conditions were the most prevalent (30%), followed by mental health conditions (28%) and headache (20%). Figure 2 shows the prevalence of number of additional LTCs within each LTC group. Apart from people with sleep disorders that had a somewhat higher prevalence of other co-occurring LTCs, the prevalence of co-occurring LTCs showed a similar pattern across LTC groups.

During the follow up period, 3,173 participants (29%) had one or more episodes of sick leave (≥ 31 days), 1,312 participants (12%) received disability pension due to musculoskeletal conditions, and 1,223 (11%) received disability pension due to all other causes. The IRs of sick leave due to musculoskeletal conditions increased with number of co-occurring LTCs, and this trend was more pronounced for disability pension (Tables 3 and 4). Specifically, the age-adjusted IRs for disability pension due to musculoskeletal conditions (per 10,000 person-years) was 87 (95% CI 75 to 98) in participants without any LTCs and 167 (95% CI 154 to 179) if they had ≥ 2 LTCs, corresponding to a HR of 1.93 (95% CI 1.66 to 2.25). People with ≥ 2 LTCs reported greater mean number of sick leave days compared to those with no LTCs during the five year follow up, i.e., 80 (95% CI 76 to 84) versus 58 (95% CI 53 to 63), respectively (Table S1 in Supplementary File 2). Similarly, those with ≥ 2 LTCs reported longer median duration of each sick leave episode (in days) compared to those with no LTCs, i.e., 75 (IQR 31 to 202) versus 64 (IQR 31 to 173), respectively.

**Table 3:** Incidence rate and relative risk of sick leave due to musculoskeletal conditions stratified by number of long-term conditions

	Person-years	No. of cases	Unadjusted IR^a^	Age-adjusted IR^a^ (95% CI)	Unadjusted HR	Age-adjusted HR (95% CI)
No LTCs	12,074	867	718	720 (672 to 768)	1.00	1.00 (reference)
1 LTC	15,995	1,344	840	843 (798 to 888)	1.17	1.17 (1.06 to 1.29)
≥ 2 LTCs	22,181	2,143	966	968 (927 to 1,009)	1.34	1.34 (1.22 to 1.47)

*LTC* Long-term condition, *IR* Incidence rate, *HR* Hazard Ratio, *CI* Confidence Interval

^a^Incidence rate per 10,000 person-years

**Table 4:** Incidence rate and relative risk of disability pension stratified by number of long-term conditions

Disability pension due to musculoskeletal conditions							Disability pension due to all cause				
	Person-years	No. of cases	Unadjusted IR^a^	Age-adjusted IR^a^ (95% CI)	Unadjusted HR	Age-adjusted HR (95% CI)	No. of cases	Unadjusted IR^a^	Age-adjusted IR^a^ (95% CI)	Unadjusted HR	Age-adjusted HR (95% CI)
No LTCs	29,867	216	72	87 (75 to 98)	1.00	1.00 (reference)	375	126	155 (130 to 160)	1.00	1.00 (reference)
1 LTC	39,016	367	94	113 (101 to 125)	1.30	1.30 (1.10 to 1.54)	694	178	206 (190 to 222)	1.42	1.42 (1.25 to 1.61)
≥ 2 LTCs	51,918	729	140	167 (154 to 179)	1.93	1.93 (1.66 to 2.25)	1,466	282	325 (308 to 342)	2.25	2.26 (2.01 to 2.53)

*LTC* Long-term condition, *IR* Incidence rate, *HR* Hazard Ratio, *CI* Confidence Interval

^a^Incidence rate per 10,000 person-years

**Fig. 3:**
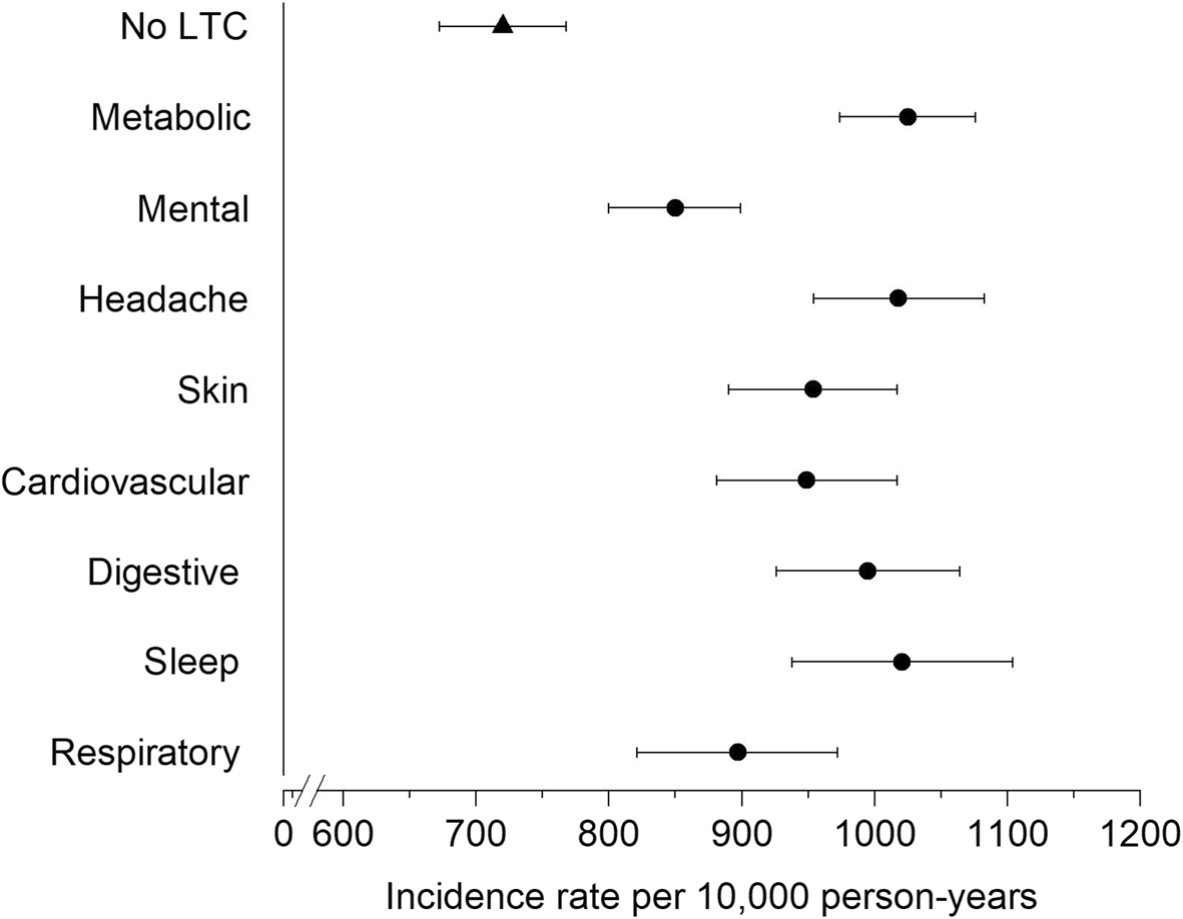
Age-adjusted incidence rate of sick leave due to musculoskeletal conditions among people with specific long-term conditions co-occurring with musculoskeletal pain

**Fig. 4:**
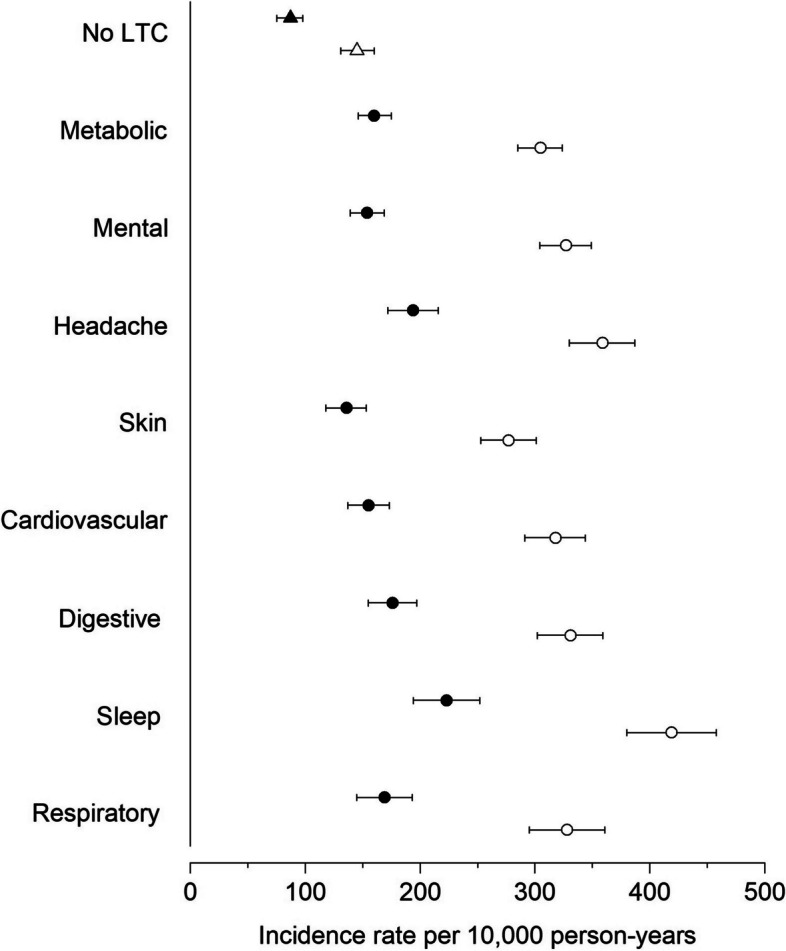
Age-adjusted incidence rate of disability pension due to musculoskeletal conditions (black filled symbols) and due to all cause (white open symbols) among people with specific long-term conditions co-occurring with musculoskeletal pain

The IRs of sick leave due to musculoskeletal conditions (per 10,000 person-years) varied only slightly according to the specific LTCs (Fig. 3, Table S2 in Supplementary File 2), with mental and behavioral conditions having the lowest IR (850, 95% CI 800 to 899). People without any LTCs had an IR of 720 (95% CI 672 to 768) and those with metabolic conditions had the highest IR (1,025, 95%CI 974 to 1,076). Sick leave results were comparable when time-to-first event analysis was used, although the IRs were smaller (Table S3 in Supplementary File 2). Similarly, small variations in IRs of disability pension due to musculoskeletal conditions were observed according to the specific LTCs (Fig. 4 and Table S4 in Supplementary File 2), although people with sleep disorders had a somewhat higher IR (223, 95% CI 194 to 252).

While the IRs of sick leave were higher in older people (i.e., > 45 years), females, and those with lower educational level (i.e., < 12 years), the HRs associated with the number of LTCs were largely similar across strata of age, sex, and educational level. This was also observed for disability pension, although the HR of disability pension associated with ≥ 2 LTCs was higher among younger than older people (HR of 2.72, 95% CI 1.92 to 3.86 vs 1.78, 95% CI 1.50 to 2.11, respectively) (Table S5-7 in Supplementary File 2).

## Discussion

In this study of working-age people with persistent musculoskeletal pain, multiple LTCs (i.e., ≥ 2 LTCs) were common, occurring in nearly half of the sample. The incidence of sick leave showed a dose-dependent association with the number of LTCs, and this trend was stronger for disability pension, i.e., the incidence of disability pension for people with musculoskeletal pain reporting ≥ 2 LTCs was twice as high compared to those with no LTCs. Moreover, people with ≥ 2 LTCs had a higher mean number of sick leave days as well as longer sick leave episodes compared to those without any LTCs. Further, the incidence of sick leave and disability pension due to musculoskeletal conditions was largely similar across groups with specific LTCs. However, people reporting sleep problems co-occurring with musculoskeletal pain had a somewhat higher incidence of disability pension.

Our study adds to the current literature showing that people with persistent musculoskeletal pain and multiple co-occurring LTCs have a substantially increased likelihood of prematurely leaving the work force due to musculoskeletal conditions compared to those without co-occurring LTCs. This increased incidence might reflect a greater burden among those with multiple LTCs, thus limiting the capacity to maintain adequate work ability. This is consistent with previous research showing a dose-dependent association between number of LTCs and work productivity loss (e.g., sick leave, early retirement) in cross-sectional [18, 19] and prospective studies [20]. Importantly, our findings are specific to sick leave and disability pension due to musculoskeletal conditions which can indicate that musculoskeletal complains are the dominating problem. However, it cannot be ruled out that musculoskeletal complains are more often registered as main diagnosis compared to other co-occurring LTCs.

Further, our findings suggest that the increased incidence of sick leave and disability pension associated with LTCs mostly depends on the number of LTCs rather than the specific LTC involved. Nonetheless, people with musculoskeletal pain reporting sleep disorders showed a somewhat high incidence of disability pension due to musculoskeletal conditions. Sleep problems are known risk factors for all-cause work disability [21, 22], and their link with work disability due to musculoskeletal pain has been previously explored [23–25]. In our sample of people with persistent musculoskeletal pain, those reporting sleep disorders had higher prevalence of co-occurring LTCs compared to other LTC groups (Fig. 2). Thus, it is possible that they represent a more vulnerable group among people with musculoskeletal pain.

Mental health conditions are, similarly to musculoskeletal conditions, one of the leading causes of work productivity loss [26–28]. Thus, it is conceivable that the co-occurrence of these conditions could result in a disproportionate high incidence of sick leave and permanent work disability compared to each condition occurring alone. Surprisingly, we found similar incidence of sick leave for people with these co-occurring conditions compared to those with only musculoskeletal pain. This is in contrast with previous studies showing greater sickness absence (in days) for the combination of musculoskeletal pain and mental health conditions, e.g., anxiety, depression compared to each condition alone [10–12]. Importantly, these studies where mostly cross-sectional, did not assess disease-specific causes of sick leave (i.e., musculoskeletal conditions) and the outcomes were based on self-report. The case definition of mental health conditions might also contribute to these differences, i.e., our definition based on the Hospital Anxiety and Depression Scale (score ≥ 8 [29]) might have detected mild cases of anxiety and depression. Further, people reporting alcohol problems were included in this LTC group. Alcohol problems have shown to be more prevalent among unemployed people [30]. As such, those who remain employed (included in this sample) might be the less severe cases with lower estimated likelihood for sick leave and disability pension. This is also corroborated by a previous study showing a small association between substance use disorders with sick leave compared to common mental health conditions such as anxiety and depression [12].

### Strengths and limitations

The strengths of this study include a large sample of participants with persistent musculoskeletal pain that allowed analyses of different LTCs while preserving relatively high precision of the estimated IRs. Further, we used information on 25 conditions covering 8 body organ systems classified according to ICD-11. A previous review suggested at least 12 LTCs to be included for prevalence studies on multimorbidity [31]. Additionally, the use of national registry data for work-related outcomes allowed us to provide risk estimates from an unbiased dataset with no lost to follow up. Finally, the use of recurrent event analysis for sick leave data enabled us to capture more fully the disease burden and provide a more precise estimation of the five-year sick leave incidence compared to time-to-first event analysis.

Some limitations need to be acknowledged. Firstly, most LTCs were classified using self-reported data. While self-report has been shown to provide reliable estimates of multimorbidity in studies with large sample [31], we cannot exclude potential misclassification. Further, while we used clinically relevant definitions and cut-offs available in the literature for defining LTCs, caution should be taken when comparing the risk estimates across LTC groups as LTCs that were categorised with more strict criteria might have led to inflated risk estimates due to greater severity of the LTC involved. Another potential limitation is representativeness of the sample [32]. A previous HUNT study comparing responders with non-responders has shown a link between people’s likelihood to participate and the prevalence of self-reported LTCs [33]. Thus, we cannot rule out that some people with specific LTCs were underrepresented, leading to potentially biased risk estimates across LTC groups. We also acknowledge the possibility of competing risk when examining work disability data, i.e., the occurrence of work disability due to musculoskeletal conditions is precluded by the earlier occurrence of work disability due to other causes (or death). If some LTC groups had higher mortality or risk of disability pension due to other causes, then the estimated incidence of disability pension due to musculoskeletal conditions would be underestimated. However, the proportion of people receiving disability pension due to musculoskeletal conditions and all (other) causes was similar across groups (Supplementary File 2: Figure S1). We also present data for disability pension due to all causes that are largely similar to the disability pension due to musculoskeletal conditions. Finally, the sample may include people who are not eligible to receive work-related benefits, e.g., students. If there is a socio-economic gradient associated with the number of LTCs (i.e., those with ≥ 2 LTCs completing less years of education), then the risk estimates for the group with multiple LTCs would be overestimated in this young group.

## Conclusions

Among people with persistent musculoskeletal pain, multiple LTCs are common and are associated with increased likelihood of prematurely leaving the work force due to musculoskeletal conditions mostly irrespective of the type of LTC involved. These findings highlight the importance of LTCs when evaluating work outcomes related to musculoskeletal health conditions, suggesting that people with musculoskeletal pain and multiple co-occurring LTCs should receive particular attention to maintain long-term work participation.

### Supplementary Information

**Supplementary Material 1. **

**Supplementary Material 2. **

## Data Availability

To protect participants’ privacy, HUNT Research Centre aims to limit storage of data outside HUNT databank and cannot deposit data in open repositories. HUNT databank has precise information on data exported to this project and are able to reproduce these upon request. There are no restrictions regarding data export given approval of applications to HUNT Research Centre. For more information see: http://www.ntnu.edu/hunt/data or contact the HUNT Study administration at kontakt@hunt.ntnu.no. Data from the National Insurance Database on sickness and disability benefits are not available due to ethical approval.

## Abbreviations

LTC: Long-term condition
IR: Incidence ratio
HR: Hazard ratio
IQR: Interquartile range
ICD: International Classification of Diseases
ICPC: International Classification of Primary Care

## References

[1] Vos T, Lim SS, Abbafati C, Abbas KM, Abbasi M, Abbasifard M (2020). Global burden of 369 diseases and injuries in 204 countries and territories, 1990–2019: a systematic analysis for the Global Burden of Disease Study 2019. Lancet.

[2] Briggs AM, Cross MJ, Hoy DG, Sànchez-Riera L, Blyth FM, Woolf AD (2016). Musculoskeletal health conditions represent a global threat to healthy aging: a report for the 2015 World Health Organization world report on ageing and health. Gerontologist.

[3] Ihlebaek C, Laerum E (2010). [Hits most, costs most and gets least]. Tidsskr Nor Laegeforen.

[4] Kinge JM, Sælensminde K, Dieleman J, Vollset SE, Norheim OF (2017). Economic losses and burden of disease by medical conditions in Norway. Health Policy.

[5] Cancelliere C, Donovan J, Stochkendahl MJ, Biscardi M, Ammendolia C, Myburgh C (2016). Factors affecting return to work after injury or illness: best evidence synthesis of systematic reviews. Chiropr Man Ther.

[6] Lowe DB, Taylor MJ, Hill SJ (2017). Associations between multimorbidity and additional burden for working-age adults with specific forms of musculoskeletal conditions: a cross-sectional study. BMC Musculoskelet Disord.

[7] Søndergaard E, Willadsen TG, Guassora AD, Vestergaard M, Tomasdottir MO, Borgquist L (2015). Problems and challenges in relation to the treatment of patients with multimorbidity: general practitioners’ views and attitudes. Scand J Prim Health Care.

[8] Wallace E, Salisbury C, Guthrie B, Lewis C, Fahey T, Smith SM (2015). Managing patients with multimorbidity in primary care. BMJ.

[9] Lowe D, Taylor M, Hill S (2016). Changing definitions altered multimorbidity prevalence, but not burden associations, in a musculoskeletal population. J Clin Epidemiol.

[10] Demyttenaere K, Bonnewyn A, Bruffaerts R, Brugha T, De Graaf R, Alonso J (2006). Comorbid painful physical symptoms and depression: prevalence, work loss, and help seeking. J Affect Disord.

[11] Beales D, Kyaw-Myint S, Smith A, O’Sullivan P, Pransky G, Linton S (2017). Work productivity loss in young workers is substantial and is associated with spinal pain and mental ill-health conditions. J Occup Environ Med.

[12] Buist-Bouwman M, de Graaf R, Vollebergh W, Ormel J (2005). Comorbidity of physical and mental disorders and the effect on work‐loss days. Acta Psychiatr Scand.

[13] Dorner T, Alexanderson K, Svedberg P, Tinghög P, Ropponen A, Mittendorfer-Rutz E (2016). Synergistic effect between back pain and common mental disorders and the risk of future disability pension: a nationwide study from Sweden. Psychol Med.

[14] Åsvold BO, Langhammer A, Rehn TA, Kjelvik G, Grøntvedt TV, Sørgjerd EP (2022). Cohort profile update: the HUNT study. Norway. Int J Epidemiol..

[15] Marcuzzi A, Nielsen TIL, Aasdahl L, Skarpsno E, Mork PJ, Nordstoga AL. Better health for people with musculoskeletal pain: the importance of multimorbidity, socioeconomic status, and family history of pain. 2023. 10.17605/OSF.IO/QEV97.

[16] Kuorinka I, Jonsson B, Kilbom A, Vinterberg H, Biering-Sørensen F, Andersson G (1987). Standardised nordic questionnaires for the analysis of musculoskeletal symptoms. Appl Ergon.

[17] Vinjerui KH, Bjerkeset O, Bjorngaard JH, Krokstad S, Douglas KA, Sund ER (2020). Socioeconomic inequalities in the prevalence of complex multimorbidity in a Norwegian population: findings from the cross-sectional HUNT study. BMJ Open.

[18] Schofield DJ, Shrestha RN, Passey ME, Earnest A, Fletcher SL (2008). Chronic disease and labour force participation among older australians. Med J Aust.

[19] Fouad AM, Waheed A, Gamal A, Amer SA, Abdellah RF, Shebl FM (2017). Effect of chronic diseases on work productivity. J Occup Environ Med.

[20] Troelstra SA, Straker LM, Harris M, Brown S, van der Beek AJ, Coenen P (2020). Multimorbidity is common among young workers and related to increased work absenteeism and presenteeism. Scand J Work Environ Health.

[21] Sivertsen B, Øverland S, Pallesen S, Bjorvatn B, Nordhus IH, Maeland JG (2009). Insomnia and long sleep duration are risk factors for later work disability. The Hordaland Health Study. J Sleep Res.

[22] Eriksen W, Natvig B, Bruusgaard D (2001). Sleep problems: a predictor of long-term work disability? A four-year prospective study. Scand J Public Health.

[23] Salo P, Oksanen T, Sivertsen B, Hall M, Pentti J, Virtanen M (2010). Sleep disturbances as a predictor of cause-specific work disability and delayed return to work. Sleep.

[24] Lallukka T, Haaramo P, Lahelma E, Rahkonen O (2011). Sleep problems and disability retirement: a register-based follow-up study. Am J Epidemiol.

[25] Jansson C, Alexanderson K, Kecklund G, Åkerstedt T (2013). Clinically diagnosed insomnia and risk of all-cause and diagnosis-specific disability pension: a nationwide cohort study. Sleep Disorders.

[26] Theis KA, Roblin DW, Helmick CG, Luo R (2018). Prevalence and causes of work disability among working-age US adults, 2011–2013, NHIS. Disability and health journal.

[27] Investigators EM, Alonso J, Angermeyer M, Bernert S, Bruffaerts R, Brugha T (2004). Disability and quality of life impact of mental disorders in Europe: results from the European study of the Epidemiology of Mental disorders (ESEMeD) project. Acta Psychiatr Scand.

[28] De Graaf R, Tuithof M, Van Dorsselaer S, Ten Have M (2012). Comparing the effects on work performance of mental and physical disorders. Soc Psychiatry Psychiatr Epidemiol.

[29] Bjelland I, Dahl AA, Haug TT, Neckelmann D (2002). The validity of the hospital anxiety and depression scale: an updated literature review. J Psychosom Res.

[30] Henkel D (2011). Unemployment and substance use: a review of the literature (1990–2010). Curr Drug Abuse Rev.

[31] Fortin M, Stewart M, Poitras M-E, Almirall J, Maddocks H (2012). A systematic review of prevalence studies on multimorbidity: toward a more uniform methodology. Annals Family Med.

[32] Fox MP, Murray EJ, Lesko CR, Sealy-Jefferson S (2022). On the need to revitalize descriptive epidemiology. Am J Epidemiol.

[33] Langhammer A, Krokstad S, Romundstad P, Heggland J, Holmen J (2012). The HUNT study: participation is associated with survival and depends on socioeconomic status, diseases and symptoms. BMC Med Res Methodol.

