# Supplementary material for: Incidence of sick leave and disability pension in adults with musculoskeletal pain and co-occurring long-term conditions: data from the Norwegian HUNT study and national registries
Source: BMC Musculoskelet Disord. 2024 Apr 8;25:273. doi: 10.1186/s12891-024-07405-1 (PMC11003184; doi:10.1186/s12891-024-07405-1)
Supplement: Supplementary file 1 — Supplementary Material 1. [file 12891_2024_7405_MOESM1_ESM.docx]

**Supplementary file 1**

**Categorisation of long-term conditions from HUNT3 data according to the International Classification of Diseases (ICD-11) based on [1]**

1. Endocrine, nutritional, or metabolic diseases (Chapter V)
   1. **Diabetes** was assessed using the following question: *“Have you had, or do you have diabetes?”*. Those who answered ‘yes’ to the question were categorised as having diabetes.
   2. **Obesity** was assessed using the measurements of weight and height and constructing the body mass index (BMI) variable as weight (kg)/height (m)^2^. Obesity was defined as BMI ≥ 30 kg/m^2^.
   3. **Hypercholesterolemia** was assessed using the measurement of total cholesterol. Those who had total cholesterol ≥8 mmol/L were defined as having hypercholesterolemia.
   4. **Thyroidal dysfunction** was assessed using the following question: *“Has it ever been verified that you have/have had hypothyroidism?”* or *“Has it ever been verified that you have/have had hyperthyroidism?”*. Those who answered ‘yes’ to either of the two questions were categorised as having thyroidal disfunction.
2. Mental or behavioural disorders (Chapter VI)
   1. **Anxiety and depression** were assessed by the Hospital Anxiety and Depression Scale [2]. This is a 14-item scale whereby 7 items assess anxiety symptoms (HADS-A), and 7 items assess depressive symptoms (HADS-D). Each item is scored on a scale from 0-3. Anxiety was defined as a score of ≥ 8 out of 21 on HADS-A, and depression was defined as a score of ≥ 8 out of 21 on HADS-D [2].
   2. **Psychiatric impairment** was assessed with the following question: *“Have you had, or do you have any of the following: impairment due to mental health problems”*, with follow up question: *“Would you describe your impairment as slight, moderate or severe?”* Those who replied having a moderate or severe impairment were categorised as having psychiatric impairment.
   3. **Alcohol use disorder** was assessed using the CAGE questionnaire [3]. This is a 4-item scale with score options of 0-1 and total score ranging from 0 to 4. Alcohol use disorders was defined as a score of ≥ 2.
3. Sleep-wake disorders (Chapter VII)
   1. **Chronic insomnia symptoms** were assessed using four questions: *“How often in the last 3 months have you:*

*1) had difficulty falling asleep?*

*2) woken up repeatedly during the night?*

*3) woken too early and couldn’t get back to sleep?*

*4) felt sleepy during the day?”*

Response options for all four questions were never/seldom, sometimes, several times a week. Chronic insomnia was defined as answering several times a week to any of the nighttime symptoms (1-3 questions) and several times a week to question 4. This classification approximates insomnia diagnosis according to the current diagnostic classification system [4].

- 1. **Sleep apnoea** was assessed with the following question: *“How often in the last 3 months have you stopped breathing when you were sleeping”* with response options never/seldom, sometimes, several times a week. Those who answered several times a week were categorised as having sleep apnoea.

1. Diseases of the nervous system (Chapter VIII)
   1. **Chronic headache** was assessed with the following question: *“Have you had headaches in the last year?”*. Those who answered ‘yes’ had follow up questions:

*1) “What type of headache?”* with response options: migraine, other headaches; and

*2) “Average number of days a month with headaches”* with response options less than 1 day, 1-6 days, 7-14 days, more than 14 days.

Chronic headache was defined as having headaches other than migraine that lasted more than 14 days per month [5].

- 1. **Migraine** was assessed with the question *“Have you had headaches in the last year?”*. Those who answered ‘yes’ had follow up questions:

- *“What is the average strength of your headache?”* with response options mild, moderate, strong

- *“How long does the headache usually last?”* with response options less than 4 hours, 4 hours – 1 day, 1 to 3 days, more than 3 days

- *“Are the headaches usually characterised or accompanied by:*

*1) throbbing/thumping pain*

*2) pain on one side of the head*

*3) worsening with physical activity*

*4) nausea and/or vomiting*

*5) hypersensitivity to light and/or noise*

Those who answered having headache lasting 0 to 72 hours with at least two of four characteristics (i.e., throbbing/thumping pain, unilateral pain, moderate/strong intensity, worsening with physical activity) and during the headache having at least one of the two symptoms (nausea and/or vomiting or hypersensitivity to light and/or noise) were defined as having migraine [5].

- 1. **Epilepsy** was assessed with the question: *“Do you have, or have you ever had epilepsy?”.* Those who answered ‘yes’ were defined as having epilepsy.

1. Diseases of the circulatory system (Chapter XI)
   1. **Myocardial infarction** was assessed with the following question: *“Do you have, or have you had myocardial infarction?”*. Those who answered ‘yes’ were categorised as having myocardial infarction.
   2. **Angina pectoris** was assessed with the following question: *“Do you have, or have you had angina pectoris?”*. Those who answered ‘yes’ were categorised as having angina pectoris.
   3. **Heart failure** was assessed with the following question: *“Do you have, or have you had heart failure?”*. Those who answered ‘yes’ were categorised as having heart failure.
   4. Other heart diseases were assessed with the following questions: *“Do you have, or have you had other heart diseases?”*. Those who answered ‘yes’ were categorised as having other heart diseases.
   5. **Hypertension** was assessed using the measurement of blood pressure. The mean of measurement 2 and 3 was calculated by the HUNT Databank. Hypertension was defined as mean systolic blood pressure of ≥180 mmHg or mean diastolic blood pressure of ≥110 mmHg or reporting using antihypertensive medications.
2. Diseases of the respiratory system (Chapter XII)
   1. **Chronic bronchitis, emphysema or chronic obstructive pulmonary disease** (COPD) were assessed with the question: *“Do you have, or have you ever had chronic bronchitis, emphysema or COPD?”.* Those who answered ‘yes’ were defined as having any of these conditions.
   2. **Asthma** was assessed with the question: *“Do you have, or have you ever had asthma?”.* Those who answered ‘yes’ were defined as having asthma.
3. Diseases of the digestive system
   1. **Irritable bowel syndrome** was assessed with the question: *“Have you had stomach pain or discomfort in the last 12 months?”.* Response options were yes much, yes a little, no. Those who answered ‘yes’ had follow up questions:

*1) “In the last 3 months, have you had this often as 1 day a week for at least 3 weeks?*

*2) “Is the pain/discomfort relieved by having a bowel movement?”*

*3) “Is the pain/discomfort related to more frequent or less frequent bowel movements than normal”*

*4) “Is the pain/discomfort related to the stool being softer or harder than usual?”*

Irritable bowel syndrome was defined as having little or much stomach pain/discomfort in the last year as often as 1 day a week for at least 3 weeks with at least two of the characteristics listed in 2-4 questions. This classification approximates the modified version of the Rome criteria for diagnosing irritable bowel syndrome [6].

- 1. **Gastro-oesophageal reflux** was assessed with the question: *“To what degree have you had the following problems in the last 12 months?”*. Response options included heartburn/acid regurgitation, and frequency options were never, a little, much. Gastro-oesophageal reflux was defined as having much heartburn/acid regurgitation in the last 12 months.

1. Diseases of the skin (Chapter XIV)
   1. **Psoriasis** was assessed with the question: *“Do you have, or have you ever had psoriasis?”.* Those who answered ‘yes’ were defined as having psoriasis.
   2. **Hand eczema** was assessed with the question: *“Do you have, or have you ever had psoriasis?”.* Those who answered ‘yes’ were defined as having hand eczema.

**Missing data**

|  | Missing (% total) |
| --- | --- |
| 1a. Anxiety based on Hospital Anxiety and Depression Scale | 283 (2.6) |
| 1a. Depression based on Hospital Anxiety and Depression Scale | 286 (2.6) |
| 1b. Psychiatric impairment* | 0 (0.0) |
| 1c. Alcohol use disorder | 957 (8.6) |
| 2a. Insomnia symptoms | 206 (1.9) |
| 2b. Sleep apnoea | 252 (2.3) |
| 3a. Diabetes | 1 (0.0) |
| 3b. Obesity | 13 (0.1) |
| 3c. Hypercholesterolemia | 249 (2.2) |
| 3d. Thyroid disfunctions | 280 (2.5) |
| 4a. Myocardial infarction | 1 (0.0) |
| 4b. Angina | 1 (0.0) |
| 4c. Heart failure | 1 (0.0) |
| 4d. Other heart diseases | 1 (0.0) |
| 4e. Hypertension | 15 (0.1) |
| 5a. Chronic headache | 157 (1.4) |
| 5b. Migraine | 157 (1.4) |
| 5c. Epilepsy | 213 (1.9) |
| 6a. Chronic bronchitis, emphysema or COPD | 1 (0.0) |
| 6b. Asthma | 1 (0.0) |
| 7a. Irritable bowel syndrome | 90 (0.8) |
| 7b. Gastro-oesophageal reflux | 521 (4.7) |
| 8a. Hand eczema | 0 (0.0) |
| 8b. Psoriasis | 3 (0.0) |

* Not possible to estimate missing data as only those with impairment were asked to answer to the question

COPD: Chronic Obstructive Pulmonary Disease

**References**

1. Vinjerui, K.H., et al., *Socioeconomic inequalities in the prevalence of complex multimorbidity in a Norwegian population: findings from the cross-sectional HUNT Study.* BMJ open, 2020. **10**(6): p. e036851.

2. Bjelland, I., et al., *The validity of the Hospital Anxiety and Depression Scale: an updated literature review.* Journal of psychosomatic research, 2002. **52**(2): p. 69-77.

3. Ewing, J.A., *Detecting alcoholism: the CAGE questionnaire.* Jama, 1984. **252**(14): p. 1905-1907.

4. Riemann, D., et al., *European guideline for the diagnosis and treatment of insomnia.* Journal of sleep research, 2017. **26**(6): p. 675-700.

5. Hagen, K., et al., *The validity of questionnaire-based diagnoses: The third Nord-Trøndelag Health Study 2006–2008.* The journal of headache and pain, 2010. **11**(1): p. 67-73.

6. Hammer, J. and N.J. Talley, *Diagnostic criteria for the irritable bowel syndrome.* The American journal of medicine, 1999. **107**(5): p. 5-11.
