# Supplementary material for: Incidence of sick leave and disability pension in adults with musculoskeletal pain and co-occurring long-term conditions: data from the Norwegian HUNT study and national registries
Source: BMC Musculoskelet Disord. 2024 Apr 8;25:273. doi: 10.1186/s12891-024-07405-1 (PMC11003184; doi:10.1186/s12891-024-07405-1)
Supplement: Supplementary file 2 — Supplementary Material 2. [file 12891_2024_7405_MOESM2_ESM.docx]

**Supplementary file 2**

Table S1: Cumulative sick leave days by number of LTCs

Table S2: Incidence rate and relative risk of sick leave due to musculoskeletal conditions by specific long-term conditions (LTCs)

Table S3: Incidence rate and relative risk of sick leave due to musculoskeletal conditions by specific long-term conditions (time to first sick leave event)

Table S4: Incidence rate and relative risk of disability pension due to musculoskeletal conditions and due to all causes by specific long-term conditions (LTCs)

Table S5: Incidence rate and relative risk of sick leave and disability pension due to musculoskeletal conditions by number of LTCs stratified by age

Table S6: Incidence rate and relative risk of sick leave and disability pension due to musculoskeletal conditions by number of LTCs stratified by sex

Table S7: Incidence rate and relative risk of sick leave and disability pension due to musculoskeletal conditions by number of LTCs stratified by educational level

Figure S1: Proportion of people receiving disability pension due to musculoskeletal conditions and all other causes stratified by LTCs

Table S1 Cumulative sick leave days (5 year follow-up) stratified by number of long-term conditions

|  |  | **Sick leave due to musculoskeletal conditions** | | | | | **Sick leave due to all causes** | |
| --- | --- | --- | --- | --- | --- | --- | --- | --- |
|  | Total n. people | N. getting sick leave (%) | Total n. sick leave days | N. episodes ≥31 days^a^ | Median (IQR) duration sick leave episodes (in days) | Mean number of sick leave days (95% CI) | Total n. sick leave days | Mean number of sick leave days  (95% CI) |
| No LTCs | 2,588 | 848 (33) | 150,131 | 950 | 64 (31 to 173) | 58 (53 to 63) | 258,759 | 100 (94 to 106) |
| 1 LTC | 3,496 | 1,261 (36) | 237,186 | 1,450 | 70 (31 to 189) | 68 (63 to 72) | 421,470 | 121 (115 to 126) |
| ≥ 2 LTCs | 4,451 | 1,994 (45) | 399,223 | 2,342 | 75 (31 to 202) | 80 (76 to 84) | 751,952 | 151 (145 to 156) |

LTC: Long-term condition; IQR: Interquartile Range; CI: confidence interval

^a^Participants can have more than one episode

Table S2 Incidence rate and relative risk of sick leave due to musculoskeletal conditions by specific long-term conditions

|  | Person-years | No. of episodes^a^ | Unadjusted IR^b^ | Age-adjusted IR^b^  (95% CI) | Unadjusted HR | Age-adjusted  HR (95% CI) |
| --- | --- | --- | --- | --- | --- | --- |
| No LTCs | 13,900 | 867 | 744 | 720 (672 to 768) | 1.00 | 1.00 (reference) |
| Metabolic | 14,900 | 1,543 | 1,036 | 1,025 (974 to 1,076) | 1.44 | 1.42 (1.29 to 1.57) |
| Mental & behavioural | 13,608 | 1,141 | 839 | 850 (800 to 899) | 1.17 | 1.17 (1.06 to 1.31) |
| Headache | 10,002 | 979 | 979 | 1,018 (954 to 1,083) | 1.36 | 1.41 (1.27 to 1.57) |
| Skin | 9,143 | 862 | 943 | 954 (890 to 1,017) | 1.31 | 1.32 (1.18 to 1.48) |
| Cardiovascular | 7,793 | 788 | 1,011 | 949 (881 to 1,017) | 1.40 | 1.32 (1.17 to 1.48) |
| Digestive | 8,094 | 801 | 990 | 995 (926 to 1,064) | 1.38 | 1.38 (1.23 to 1.54) |
| Sleep | 5,783 | 580 | 1,003 | 1,021 (938 to 1,104) | 1.39 | 1.41 (1.25 to 1.60) |
| Respiratory | 6,170 | 543 | 880 | 897 (821 to 972) | 1.22 | 1.24 (1.09 to 1.41) |

LTC: Long-term condition; IR: Incidence rate; HR: Hazard ratio; CI: Confidence Interval

^a^Participants can have more than one episode

^b^Incidence rate per 10,000 person-years

Table S3 Incidence rate and relative risk of sick leave due to musculoskeletal conditions by specific long-term conditions (time to first sick leave event)

|  | Person-years | No. of cases | Age-adjusted IR^a^  (95% CI) | Age-adjusted  HR (95% CI) |
| --- | --- | --- | --- | --- |
| No LTCs | 10,610 | 642 | 606 (559 to 653) | 1.00 (reference) |
| Metabolic | 12,254 | 1,098 | 860 (809 to 911) | 1.41 (1.28 to 1.55) |
| Mental & behavioural | 11,883 | 834 | 710 (662 to 759) | 1.17 (1.05 to 1.29) |
| Headache | 8,546 | 700 | 850 (786 to 913) | 1.39 (1.25 to 1.55) |
| Skin | 7,842 | 616 | 793 (730 to 856) | 1.30 (1.16 to 1.45) |
| Cardiovascular | 6,632 | 563 | 795 (728 to 863) | 1.30 (1.16 to 1.46) |
| Digestive | 6,839 | 579 | 850 (781 to 919) | 1.39 (1.24 to 1.56) |
| Sleep | 4,916 | 412 | 853 (771 to 936) | 1.40 (1.23 to 1.60) |
| Respiratory | 5,318 | 395 | 754 (680 to 829) | 1.24 (1.09 to 1.40) |

LTC: Long-term condition; HR: Hazard Ratio

^a^Incidence rate per 10,000 person-years

Table S4 Incidence rate and relative risk of disability pension due to musculoskeletal conditions and due to all causes by specific long-term conditions

|  | **Disability pension due to musculoskeletal conditions** | | | | | | **Disability due to all causes** | | | |
| --- | --- | --- | --- | --- | --- | --- | --- | --- | --- | --- |
|  | Person-years | No. of cases | Unadjusted IR^a^ | Age-adjusted IR^a^  (95% CI) | Unadjusted HR | Age-adjusted  HR (95% CI) | Person-years | No. of cases | Age-adjusted IR^a^  (95% CI) | Age-adjusted  HR (95% CI) |
| No LTCs | 29,867 | 216 | 72 | 87 (75 to 98) | 1.00 | 1.00 (reference) | 29,867 | 375 | 145 (131 to 160) | 1.00 (reference) |
| Metabolic | 34,377 | 492 | 143 | 160 (146 to 175) | 1.96 | 1.86 (1.58 to 2.18) | 34,377 | 956 | 305 (285 to 324) | 2.11 (1.87 to 2.38) |
| Mental & behavioural | 32,634 | 400 | 123 | 154 (139 to 169) | 1.69 | 1.78 (1.51 to 2.10) | 32,634 | 882 | 327 (304 to 349) | 2.26 (2.00 to 2.55) |
| Headache | 24,902 | 336 | 135 | 194 (172 to 216) | 1.86 | 2.22 (1.87 to 2.64) | 24,902 | 662 | 359 (330 to 387) | 2.47 (2.17 to 2.80) |
| Skin | 22,506 | 245 | 109 | 136 (118 to 153) | 1.50 | 1.57 (1.30 to 1.88) | 22,506 | 521 | 277 (253 to 301) | 1.91 (1.67 to 2.18) |
| Cardiovascular | 15,451 | 278 | 180 | 155 (137 to 173) | 2.44 | 1.80 (1.51 to 2.16) | 15,451 | 560 | 318 (291 to 344) | 2.23 (1.95 to 2.54) |
| Digestive | 18,910 | 273 | 144 | 176 (155 to 197) | 1.98 | 2.03 (1.70 to 2.43) | 18,910 | 532 | 331 (302 to 359) | 2.29 (2.01 to 2.61) |
| Sleep | 13,506 | 234 | 173 | 223 (194 to 252) | 2.38 | 2.57 (2.14 to 3.10) | 13,506 | 460 | 419 (380 to 458) | 2.91 (2.54 to 3.34) |
| Respiratory | 14,881 | 196 | 132 | 169 (145 to 193) | 1.81 | 1.95 (1.61 to 2.37) | 14,881 | 397 | 328 (295 to 361) | 2.27 (1.97 to 2.61) |

LTC: Long-term condition; IR: Incidence rate; HR: Hazard ratio; CI: Confidence Interval

^a^Incidence rate per 10,000 person-years

Table S5 Incidence rate and relative risk of sick leave and disability pension by numbers of long-term conditions stratified by age

|  | **Sick leave due to musculoskeletal conditions** | | | |  | **Disability pension due to musculoskeletal conditions** | | | |
| --- | --- | --- | --- | --- | --- | --- | --- | --- | --- |
|  | Person-years | No. of episodes | Unadjusted IR^a^  (95% CI) | Unadjusted HR (95% CI) |  | Person-years | No. of cases | Unadjusted IR^a^  (95% CI) | Unadjusted HR (95% CI) |
| **≤ 45 years** |  |  |  |  |  |  |  |  |  |
| No LTCs | 4,530 | 264 | 583 (517 to 657) | 1.00 (reference) |  | 13,222 | 38 | 29 (21 to 40) | 1.00 (reference) |
| 1 LTC | 6,173 | 464 | 752 (686 to 823) | 1.29 (1.08 to 1.54) |  | 17,819 | 82 | 46 (37 to 57) | 1.61 (1.10 to 2.37) |
| ≥ 2 LTCs | 8,351 | 714 | 855 (795 to 920) | 1.47 (1.24 to 1.73) |  | 23,424 | 180 | 77 (66 to 89) | 2.72 (1.92 to 3.86) |
| **> 45 years** |  |  |  |  |  |  |  |  |  |
| No LTCs | 7,544 | 603 | 799 (738 to 866) | 1.00 (reference) |  | 16,645 | 178 | 107 (92 to 124) | 1.00 (reference) |
| 1 LTC | 9,822 | 880 | 896 (839 to 957) | 1.09 (0.98 to 1.23) |  | 21,197 | 285 | 135 (120 to 151) | 1.25 (1.04 to 1.51) |
| ≥ 2 LTCs | 13,830 | 1,429 | 1,033 (981 to 1,088) | 1.28 (1.14 to 1.42) |  | 28,494 | 549 | 193 (177 to 210) | 1.78 (1.50 to 2.11) |

LTC: Long-term condition; IR: Incidence rate; HR: Hazard ratio; CI: Confidence Interval

^a^Incidence rate per 10,000 person-years

Table S6 Incidence rate and relative risk of sick leave and disability pension by number of long-term conditions stratified by sex

|  | **Sick leave due to musculoskeletal conditions** | | | |  | **Disability pension due to musculoskeletal conditions** | | | |
| --- | --- | --- | --- | --- | --- | --- | --- | --- | --- |
|  | Person-years | No. of episodes | Age-adjusted IR^a^  (95% CI) | Age-adjusted  HR (95% CI) |  | Person-years | No. of cases | Age-adjusted IR^a^  (95% CI) | Age-adjusted  HR (95% CI) |
| **Males** |  |  |  |  |  |  |  |  |  |
| No LTCs | 5,266 | 303 | 583 (517 to 648) | 1.00 (reference) |  | 12,915 | 65 | 64 (48 to 80) | 1.00 (reference) |
| 1 LTC | 6,951 | 501 | 727 (663 to 791) | 1.25 (1.06 to 1.47) |  | 16,688 | 113 | 84 (68 to 100) | 1.32 (0.97 to 1.79) |
| ≥ 2 LTCs | 8,823 | 771 | 876 (814 to 938) | 1.50 (1.28 to 1.75) |  | 20,554 | 208 | 121 (104 to 138) | 1.91 (1.45 to 2.53) |
| **Females** |  |  |  |  |  |  |  |  |  |
| No LTCs | 8,068 | 564 | 827 (759 to 895) | 1.00 (reference) |  | 16,952 | 151 | 105 (88 to 122) | 1.00 (reference) |
| 1 LTC | 9,527 | 843 | 934 (871 to 997) | 1.13 (1.00 to 1.28) |  | 22,329 | 254 | 136 (119 to 153) | 1.30 (1.06 to 1.59) |
| ≥ 2 LTCs | 11,615 | 1,372 | 1,029 (975 to 1,083) | 1.24 (1.11 to 1.39) |  | 31,364 | 521 | 200 (182 to 217) | 1.91 (1.59 to 2.29) |

LTC: Long-term condition; IR: Incidence rate; HR: Hazard ratio; CI: Confidence Interval

^a^Incidence rate per 10,000 person-years

Table S7 Incidence rate and relative risk of sick leave and disability pension by numbers of long-term conditions stratified by educational level

|  | **Sick leave due to musculoskeletal conditions** | | | |  | **Disability pension due to musculoskeletal conditions** | | | |
| --- | --- | --- | --- | --- | --- | --- | --- | --- | --- |
|  | Person-years | No. of episodes | Age-adjusted IR^a^  (95% CI) | Age-adjusted  HR (95% CI) |  | Person-years | No. of cases | Age-adjusted IR^a^  (95% CI) | Age-adjusted  HR (95% CI) |
| **> 12 years** |  |  |  |  |  |  |  |  |  |
| No LTCs | 4,097 | 210 | 516 (446 to 585) | 1.00 (reference) |  | 10,474 | 29 | 29 (20 to 38) | 1.00 (reference) |
| 1 LTC | 4,905 | 291 | 590 (523 to 659) | 1.15 (0.93 to 1.40) |  | 12,390 | 57 | 46 (36 to 56) | 1.60 (1.01 to 2.47) |
| ≥ 2 LTCs | 5,959 | 433 | 728 (659 to 797) | 1.41 (1.16 to 1.71) |  | 14,903 | 97 | 78 (66 to 89) | 2.30 (1.52 to 3.48) |
| **≤ 12 years** |  |  |  |  |  |  |  |  |  |
| No LTCs | 7,969 | 655 | 822 (759.1 to 885) | 1.00 (reference) |  | 19,373 | 186 | 125 (106 to 143) | 1.00 (reference) |
| 1 LTC | 11,052 | 1,053 | 958 (900 to 1,016) | 1.16 (1.04 to 1.30) |  | 26,534 | 310 | 155 (136 to 173) | 1.25 (1.04 to 1.50) |
| ≥ 2 LTCs | 16,195 | 1,702 | 1,053 (1,003 to 1,103) | 1.28 (1.15 to 1.42) |  | 36,953 | 629 | 219 (200 to 238) | 1.79 (1.52 to 2.11) |

LTC: Long-term condition; IR: Incidence rate; HR: Hazard ratio; CI: Confidence Interval

^a^Incidence rate per 10,000 person-years


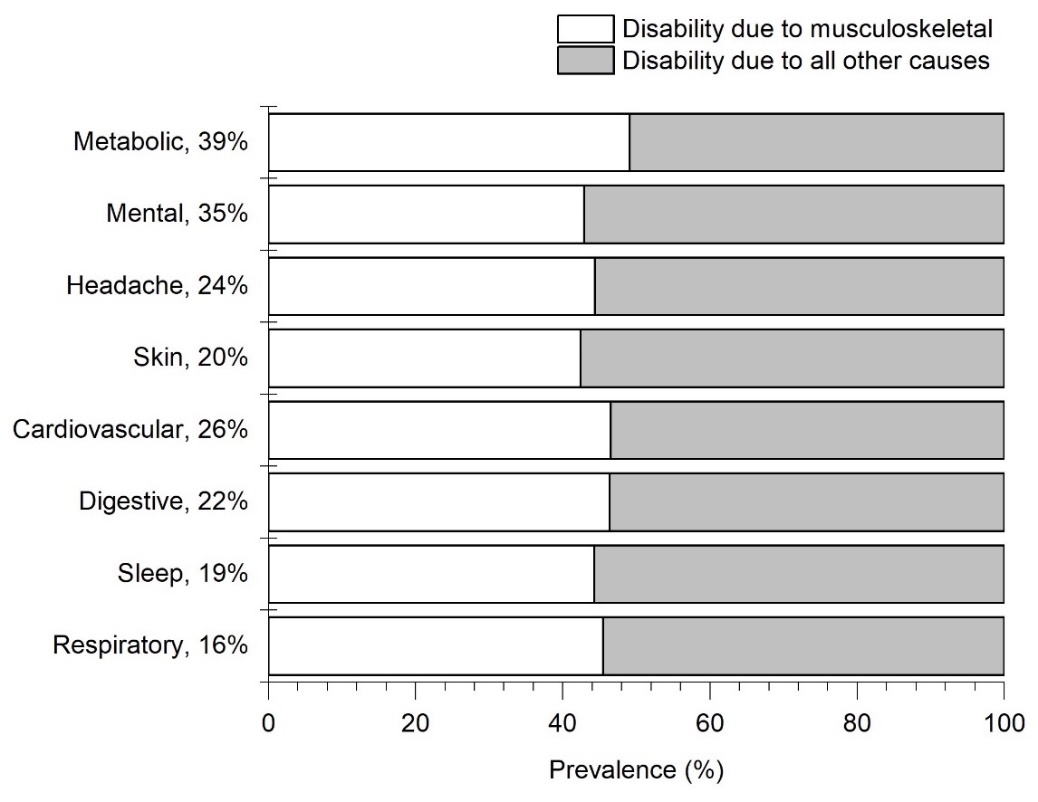


Figure S1 Proportion of people receiving disability pension due to musculoskeletal conditions and all other causes stratified by long-term conditions (LTCs). Percentages specified besides each LTC represent the proportion of people getting disability pension (any cause) for each LTC group.
